# Effects of stand age on community composition and diversity of soil bacteria and fungi in poplar plantations in Northeast China

**DOI:** 10.3389/fpls.2025.1717501

**Published:** 2025-11-21

**Authors:** Lei Zhao, Bing Mao, Hongxing Wang, Luping Jiang, Jin Huang, Zhongyi Pang, Yanhui Peng, Tongbao Qu, Xiyang Zhao

**Affiliations:** 1 College of Forestry and Grassland, Jilin Agricultural University, Changchun, China; 2 Key Laboratory of Beibu Gulf Environment Change and Resources Use, Nanning Normal University, Nanning, China; 3 Xinmin City Machinery Forest Farm, Shenyang, China

**Keywords:** microbial biomass stoichiometry, enzyme stoichiometry, bacterial community, fungal community, microbial network analysis

## Abstract

Stand age is one of the most important indicators of plantation development status after afforestation. Soil microbial community plays an essential role in ecosystem functioning. Yet, the responses of soil microbial community composition and diversity to stand development are inadequately understood. Here, we examined changes in community composition and diversity of soil bacteria and fungi in poplar plantations across stand ages and their relationships with soil chemical and biochemical properties in Northeast China. We measured soil chemical properties (organic carbon, total nitrogen, total phosphorus and their stoichiometries), soil biochemical properties (microbial biomass, soil enzyme activity and their stoichiometries), and composition and diversity of soil bacterial and fungal communities in a chronosequence (1, 4, 7 and 9 years) of poplar plantations. Furthermore, we analyzed microbial co-occurrence network and the relationships of soil bacterial and fungal community diversity and composition with soil chemical and biochemical properties. The Chao1 index of soil bacteria was lowest in the 9-year-old plantation, and Chao1 index of soil fungi was lowest in the 7-year-old plantation. Soil bacterial and fungal diversity showed a significant relationship with soil microbial biomass. The most dominant bacterial species were from *Proteobacteria*, *Acidobacteriota*, *Actinobacteriota*, *Firmicutes* and *Chloroflexi*, and fungal species were from *Ascomycota*, *Basidiomycota* and *Mortierellomycota*. The number of links and average degree of bacterial communities decreased as stand age of poplar plantations increased, while those of fungal communities increased. Soil bacterial and fungal network parameters showed significant relationship with soil microbial biomass ang microbial stoichiometry. Our results showed that the impact of stand age on soil microbial community diversity and composition is specific and stage-dependent, rather than following a simple linear trend with increasing age, and this may be due to the influence of stand age on stoichiometry of soil microbial biomass.

## 1 Introduction

Afforestation is one of the most effective means to deal with climate change. Several ecological restoration projects have been initiated by the Chinese government since the 1980s, such as converting croplands and marginal lands into forests, and Three-north Shelterbelt Project, to prevent soil and water quality degradation. Therefore, China has the largest afforested area in the world with an estimated area of 80.03 × 10^6^ ha, or 26.2% of global forest plantation area (265 × 10^6^ ha) (National Forestry and Grassland Administration, 2020). Previous studies showed that soil microbial processes are essential to the functioning of forest ecosystems, such as nutrient cycling and soil C sequestration (Tisdall, 1994; Pajares and Bohannan, 2016; Chen et al., 2013; Ni et al., 2021). During plantation management, afforestation might significantly affect the vegetation structure and soil nutrient availability by affecting soil microbial processes (Li et al., 2023; Shi et al., 2024). Consequently, understanding changes in soil microbial community and function is essential for developing management strategies designed to enhance the stability of plantation forest ecosystems.

Stand age is one of the most important indicators of plantation development status after afforestation, substantially affecting the dynamics of soil chemical and biochemical properties, enzyme activities, and microbial communities (Inagaki et al., 2004; Trogisch et al., 2016; Wang et al., 2021a; Guo and Ren, 2014). For instance, soil nutrient availability increased with increasing stand age in Chinese fir plantations (Wu et al., 2020; Xia et al., 2021). In *Pinus radiata* plantations with stand ages ranging from 1 to 33 years, soil microbial biomass N in 5-year-old was the lowest (Ross et al., 1995). Compared with 3-, 7- and 10-year-old Eucalyptus plantations, the 13-year-old plantation was found to have greater microbial biomass (Cao et al., 2010). During the development of plantations, the diversity and composition of soil microbial communities regulate most biogeochemical processes by secreting extracellular enzymes (Li et al., 2015; Qiang et al., 2020), thereby playing an essential role in influencing plant growth. Therefore, it is necessary to investigate the changes in diversity and composition of soil microbial communities during plantation development (Morriën et al., 2017).

In northern China, large-area poplar plantations have been established for wood production, urban greening, desertification control and carbon sequestration, due to their fast growth (Zhou et al., 2013). Ecologically, poplar plantations may have the potential to change the ecological environment because of their fast growth and high biomass productivity (Han et al., 2022; Song et al., 2021). However, the effects of stand ages of poplar plantations on the soil quality in the northern China and specifically on soil microbial community structure and function are poorly understood. According to previous studies, soil microbial enzymes participate in almost all soil biochemical processes and play an essential role in nutrient cycling within ecosystems (Hill et al., 2012; Song et al., 2012). Additionally, soil microbial biomass serves as a key indicator of nutrient cycling and can sensitively reflect changes in soil quality (Nannipieri et al., 1979; Moreira et al., 2011; Polyanskaya et al., 2017). Soil microbial diversity is one of the main driving factors of the soil biochemical cycle. Several studies found that afforestation greatly affects the biological and biochemical properties of soil, including soil microbial enzyme activities, microbial biomass and microbial diversity (Li et al., 2023; Shi et al., 2024). Furthermore, Co-occurrence network analyses, which reflect network complexity and interactions among bacterial and fungal species in soil microbial communities, have recently been applied in soil microbial community studies (Chen et al., 2022; Wu et al., 2023). Soil microbial network complexity is often influenced by multiple factors, such as climate change and management strategies (Tu et al., 2020; Gong et al., 2024). Several studies have confirmed that the soil physicochemical properties and disturbance levels are key factors changing structure and network topology of microbial communities (Nakayama et al., 2019; Zhu et al., 2021; Xu et al., 2022). Although microbial network analysis has been widely applied in multiple ecosystems, the understanding of how complex microbial communities respond to stand age of plantations is still inadequate.

Therefore, this study aims to investigate the responses of the soil biological properties and specifically soil microbial community structure and function to stand development (1, 4, 7 and 9 years) of poplar (*Populus cathayana × canadensis* ‘Xinlin 1’) plantations in northern China. Soil microbial diversity and community composition were determined by using amplicon high-throughput sequencing. Alpha diversity indices, including Chao1 richness, Shannon diversity, and Observed species, were calculated to assess microbial diversity within samples. Additionally, soil physicochemical properties were determined, and their relationships with soil microbial diversity, community composition, co-occurrence network pattern, and potential function were also examined. Based on the aforementioned findings, we hypothesize that: (1) soil chemical and biochemical properties and their stoichiometry were lower in the older poplar plantation (9-year-old plantation) compared with that in younger plantation (4-year-plantation or 1-year-old plantation); (2) the diversity of soil bacteria and fungi will decrease with the increase in stand ages of poplar plantation, and the complexity of the co-occurrence network of bacterial and fungal communities gradually increased with stand age; (3) the changes of soil bacterial and fungal community diversity and composition mainly related to changes in soil microbial biomass, enzyme activity and their stoichiometry rather than soil chemical properties and chemical stoichiometry.

## 2 Materials and methods

### 2.1 Experiment design

The study site was located in Xinmin State Mechanical Forest Farm (41°42′–42°17′N, 122°27′–123°20′E), Liaoning Province, China. The study site has a temperate continental monsoon climate with a mean annual temperature of 7.6 °C and precipitation of 656.5 mm. The soils are sandy loam. A chronosequence (1, 4, 7 and 9 years) of poplar (*Populus cathayana × canadansis* ‘Xinlin 1’) plantations were used in the present study.

In August 2022, five plots of 15 m × 15 m were randomly established in each stand age plantation, and the distance between adjacent plots was larger than 15 m. Four soil samples from each plot were randomly collected at 0–10 cm depth by using soil-corer with an inner diameter of 4.5 cm according to the S-shape, and then they were pooled together to give one composite sample. After stones and coarse roots were removed, soil samples were sieved (2 mm), and then divided into three sub-samples. One subsample was used to measure soil organic carbon (SOC), total nitrogen (TN), total phosphorus (TP) and pH after air dried. One subsample was stored at liquid nitrogen for later measurements of microbial community composition and diversity. Another subsample was refrigerated at 4°C and used to analyze microbial biomass, enzyme activity, NH_4_^+^ and NO_3_^-^ within two weeks.

### 2.2 Analysis of chemical and soil enzyme activity

Soil pH was measured in water extracts of 1:2.5 (*w/v*) of air-dried soil and deionized water. Soil NH_4_^+^-N and NO_3_^–^N were determined by the indophenol blue colorimetric method and ultraviolet spectrophotometry, respectively (Kempers and Zweers, 1986). After sieved, the SOC and N concentrations of soil were measured using the K_2_Cr_2_O_7_–H_2_SO_4_ wet oxidation method (Nelson and Sommers, 1996). Total N concentration was determined with a continuous-flow autoanalyzer (AutoAnalyzer III, Bran+Luebbe GmbH, Germany) after soil samples were digested with H_2_SO_4_. Total P concentration was measured with the sodium hydroxide melting-molybdenum antimony colorimetric method. The C/N, C/P and N/P ratios of soil were calculated from the values of SOC concentration and total N and P concentrations. The MBC, MBN and MBP were determined using a chloroform (CHCl_3_) based fumigation-0.5 M K_2_SO_4_ extraction method (Frey et al., 2004). The activities of four extracellular enzymes participating in the cycling of C (β-glucosidase, BG), N (N-acetyl-β-D-glucosaminidase, NAG) and P (acid phosphomonoesterase, AP) were measured following the microporous plate method (Bach et al., 2013; German et al., 2012).

### 2.3 Microbial community analysis

The total DNA of the microbial communities in the soil samples was extracted using a DNA extraction kit (E.Z.N.A ^®^ Soil DNA Kit). Three replicate PCR reactions were pooled in equal concentrations based on the PCR product yields. The PCR products were verified on a 2% agarose gel and subsequently purified using the extraction kit (E.Z.N.A ^®^ Soil DNA Kit). The purified DNA was generated from a sequencing library on the Illumina HiSeq 2500 platform (Illumina, San Diego, CA, USA). Amplification and barcoded pyrosequencing of the 16S rRNA gene were conducted according to a previously described instruction (Sengupta and Dick, 2017). The V3-V4 region of the 16S rRNA genes was amplified using the primers 515 F: GTGCCAGCMGCCGCGGTAA and 907(806)R: CCGTCAATTCCTTTGAGTT. The fungal rDNA ITS1 region was amplified using PCR with the primers ITS1F (5′-CTTGGTCATTTAGAGGAAGTAA-3′). The PCR products were sequenced using an Illumina HiSeq platform following the standard protocols. The library was constructed and sequenced on an Illumina HiSeq platform as per the standard protocols. Sequencing techniques and data analyses were also performed by Guangdong Novogene Bioinformatics Technology Co., Ltd. (Tianjin, China).

Data obtained from Illumina sequencing were handled by separating primers and adapters from the reads, and a reading of approximately 250 bp was obtained. Sequences with >97% similarity were clustered into one operational taxonomic unit (OTU) using the UPARSE algorithm (v. 7.0.1001 http://drive5.com/usearch/manual/singletons.html). The α-diversity of the bacterial community, including Shannon index and Chao1 index, was determined by OTUs using Quantitative Insights Into Bacterial Ecology (QIIME, v. 1.7.0).

### 2.4 Data analysis

Differences in chemical and biochemical properties of soil among stand ages were analyzed by one-way analysis of variance (ANOVA) according to Duncan’s test (*P* < 0.05) using the SPSS 22.0 software. For microbial diversity analysis, Shannon and Chao1 indices were used. Correlation heatmaps describing the correlations between chemical and biochemical properties of soil, soil microbial diversity and the dominant phyla, classes and genera of soil bacteria and fungi (Origin 2023). Correlation coefficients were calculated based on Pearson’s correlation coefficient method. Non-metric multidimensional scaling (NMDS) was conducted and visualized using the vegan package in R (version 4.5.1).

Response ratio (RR) was defined as the response of the soil microbial community diversity and composition to control plantation (1-year-old), and was calculated as follows (Sheng et al., 2015):


$$\text{RR}=\frac{\text{Microbial index in treatment }–(\text{Microbial index in control})}{\text{Microbial index in control}}\times 100\%$$

Network was used to explore co-occurrence patterns of abundant and rare microbial taxa at the four stand ages using the Molecular Ecological Network Analyses Pipeline (MENA, http://ieg4.rccc.ou.edu/mena) (Zhou et al., 2010; Deng et al., 2012). The similarity thresholds between 0.70 and 0.99 with 0.01 intervals were obtained and applied to the Spearman rank correlation matrix, and correlations above the specific threshold were used to compute the microbial network eigenvalues. We calculated the network topological characteristics, including average path length, nodes, edges, positive links and negative links, which were then used to describe the properties of individual nodes in the network and the overall topology of different networks. Visualization of the co-occurrence network was performed with the Gephi platform (http://gephi.github.io). Redundancy analysis (RDA) was used to determine the relative contributions of soil chemical and biochemical properties in explaining the variance of soil microbial community diversity and composition. The explanatory magnitude and significance of each explanatory variable were analyzed by hierarchical segmentation. The “Vegan” (Dixon, 2003) package in R was used for RDA.

## 3 Results

The changes in soil microbial community and function are essential for developing management strategies designed to enhance the stability of plantation forest ecosystems. Yet, the responses of soil microbial community composition and diversity to stand development of poplar plantation in northern China are inadequately understood. Therefore, the present study investigated the responses of the soil microbial diversity, community composition, co-occurrence network pattern, and potential function to stand development (1, 4, 7 and 9 years) of poplar plantations.

### 3.1 Soil chemical and biochemical properties

**Figure 1:**
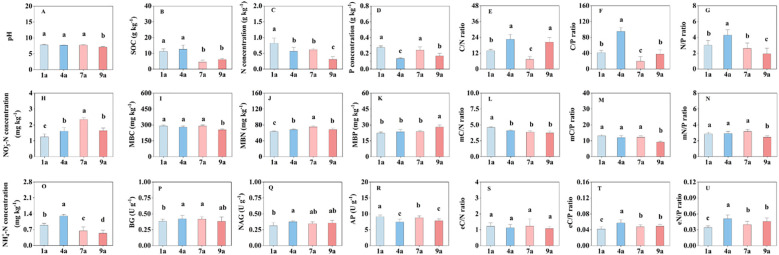
The chemical and biochemical properties of soil in different aged poplar plantations. NO_3_^–^-N, Nitrate nitrogen; NH_4_^+^-N, Ammonium nitrogen; SOC, soil organic carbon; TN, Total nitrogen; TP, Total phosphorus; C/N ratio, ratio of SOC and TN; C/P ratio, ratio of SOC and TP; N/P ratio, ratio of TN and TP; MBC, Microbial biomass carbon; MBN, Microbial biomass nitrogen; MBP, Microbial biomass phosphorus; mC/N ratio, ratio of MBC and MBN; mC/P ratio, ratio of MBC and MBP; mN/P ratio, ratio of MBN and MBP; BG, β-glucosidase; NAG, N-acetyl-β-D-glucosaminidase; AP, acid phosphomonoesterase; eC/N ratio, ratio of BG and NAG; eC/P ratio, ratio of BG and AP; eN/P ratio, ratio of NAG and AP. Different lowercase letters represent significant differences among different stand ages at *P* < 0.05, respectively.

Soil pH in the 9-year-old plantation was lower than those in the 1-, 4- and 7-year-old plantations (**Figure 1A**). There were no significant differences in soil pH among 1-, 4- and 7-year-old plantations. The SOC, N concentration and P concentration in the 1-year-old plantation were higher than those in the 9-year-old plantation (**Figures 1B–D**). There were no significant differences in soil C/N ratio between 4- and 9-year-old plantations, and soil C/N ratio was lower in the 7-year-old plantation than those in the other stand ages (**Figure 1E**). Soil C/P and N/P ratios in the 4-year-old plantation were higher than those in the other stand ages (**Figures 1E, G**). Soil NO_3_^–^N concentration in the 7-year-old plantation was higher than those in the other stand ages (**Figure 1H**). In the 1-year-old plantation, soil NO_3_^–^N concentration was lower than those in the other stand age plantations. There were no significant differences in MBC, MBP, mC/P ratio and mN/P ratio among 1-, 4- and 7-year-old plantations (**Figures 1I, K, M, N**). In the 1-year-old plantation, MBN was lower and mC/N ratio was highest than those in the other stand age plantations (**Figures 1J, L**). Soil NH_4_^+^-N concentration in the 4-year-old plantation was higher, and soil NH_4_^+^-N concentration in the 9-year-old plantation was lower than those in the other stand age plantations (**Figure 1O**). There were no significant differences in BG and NAG activities among 4-, 7- and 9-year-old plantations (**Figures 1P, Q**). Soil AP activity was higher, and eC/P and eN/P ratios were lower in the 1-year-old plantation than those in the other stand age plantations (**Figures 1E, F, R**).

### 3.2 Soil bacterial and fungal diversity and community composition

The one-way ANOVA test indicated that there were no significant differences in Shannon index of soil bacteria and fungi among four different aged poplar plantations (**Table 1**). The Chao1 index of soil bacteria in the 1-year-old plantation was higher than those in the 9-year-old plantation. There were no significant differences in Chao1 index of soil bacteria among 1-, 4- and 7-year-old plantations. The Chao1 index of soil fungi in the 1-year-old plantation was higher than those in the other three stand ages. There were no significant differences in Chao1 index of soil fungi between 4-and 7-year-old plantations.

**Table 1:** Soil microbial diversity in different aged poplar plantations.

Stand age (years)	Bacteria			Fungi		
	Shannon	Chao1	Observed species	Shannon	Chao1	Observed species
1	10.3 (0.1) ^a^	2268.2 (137.4) ^a^	2266.8 (137.7) ^a^	5.8 (0.4) ^a^	429.2 (55.7) ^a^	452.0 (42.6) ^a^
4	10.2 (0.2) ^a^	2252.8 (170.6) ^a^	2151.3 (170.2) ^b^	5.3 (0.4) ^a^	339.9 (20.9) ^b^	338.8 (20.0) ^b^
7	10.1 (0.1) ^a^	2204.2 (86.9) ^a^	2202.3 (86.5) ^a^	5.2 (0.5) ^a^	267.7 (11.4) ^c^	290.1 (41.3) ^c^
9	9.9 (0.1) ^a^	1906.5 (72.2) ^b^	1905 (71.9) ^c^	5.2 (0.8) ^a^	339.5 (34.6) ^b^	320.6 (44.9) ^b^

The data are shown as the means ± SE (*n* = 4). Different lowercase letters represent significant differences among different stand ages at *P* < 0.05.

**Figure 2:**
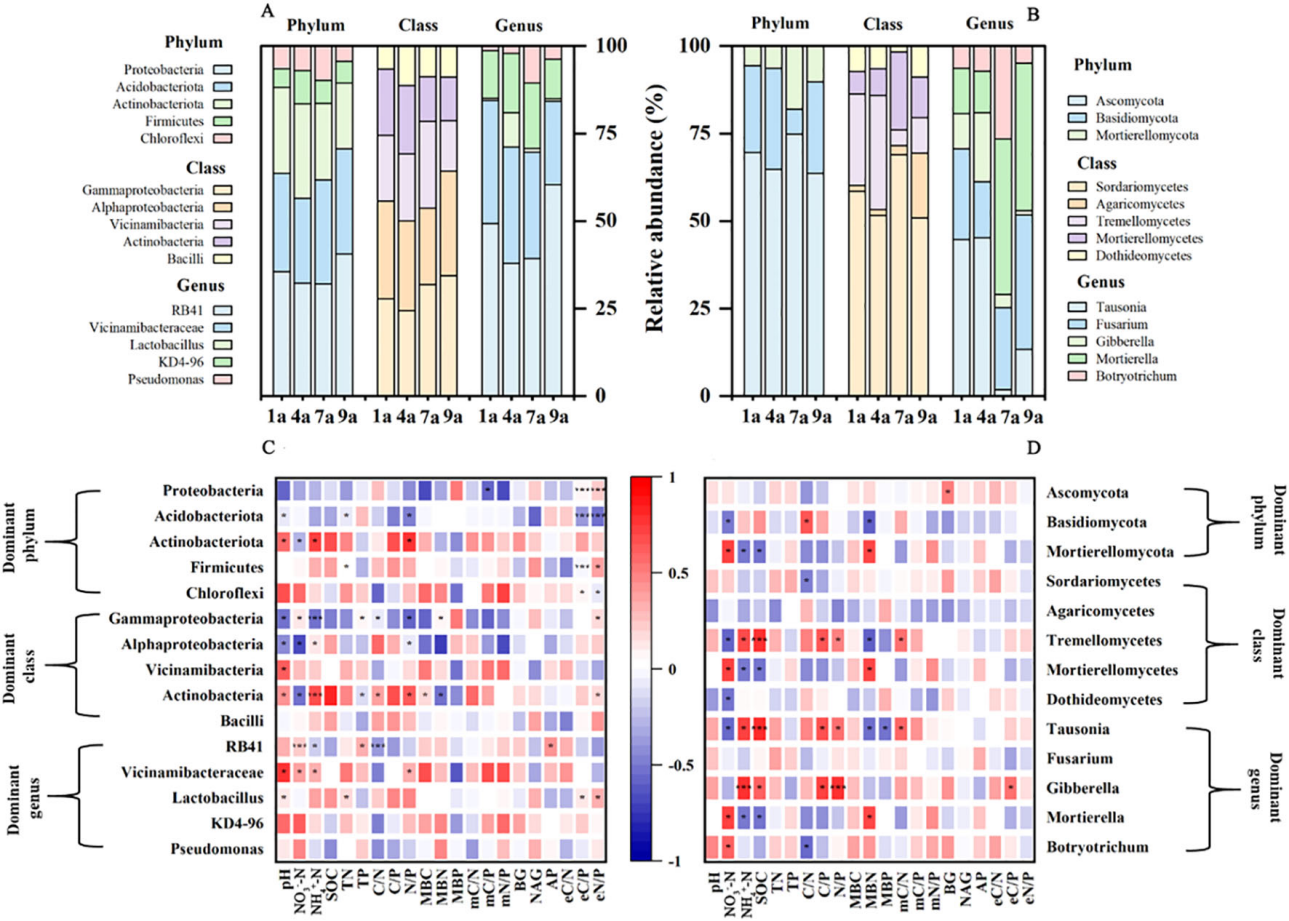
Relative abundance of the dominant phyla, classes and genera of bacteria **(A)** and fungi **(B)** in soil in different aged poplar plantations. Correlations between soil chemical and biochemical properties and dominant and keystone associations of bacteria **(C)** and fungi **(D)** at levels of phyla, classes and genera. *P < 0.05; **P < 0.01; ***P < 0.001.

**Figure 3:**
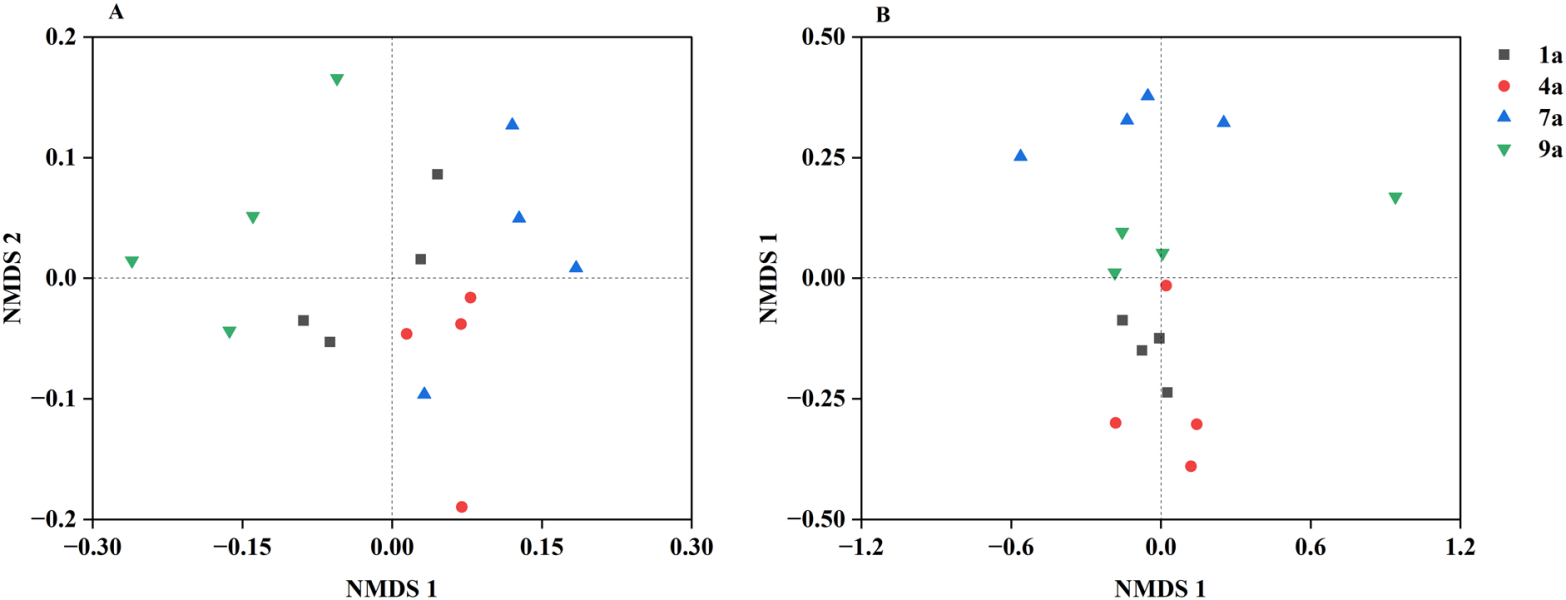
Nonmetric multidimensional scaling (NMDS) representation of soil bacterial community **(A)** and fungal community **(B)** in different aged poplar plantations.

All of the 16S reads obtained from soils of 1-, 4-, 7-year-old plantations were classified into 47 bacterial phyla, 124 classes and 726 genera (**Figures 2A**, **3A**). Overall, the phylum *Proteobacteria* dominated the bacterial communities, representing 27.02%, 25.01%, 23.82% and 30.13% in the 1-, 4-, 7- and 9-year-old plantations, respectively. All of the ITS reads obtained from soils of 1-, 4-, 7-year-old plantations were classified into 10 fungal phyla, 29 classes and 244 genera (**Figures 2B**, **3B**).

### 3.3 Soil bacterial and fungal network complexity

**Figure 4:**
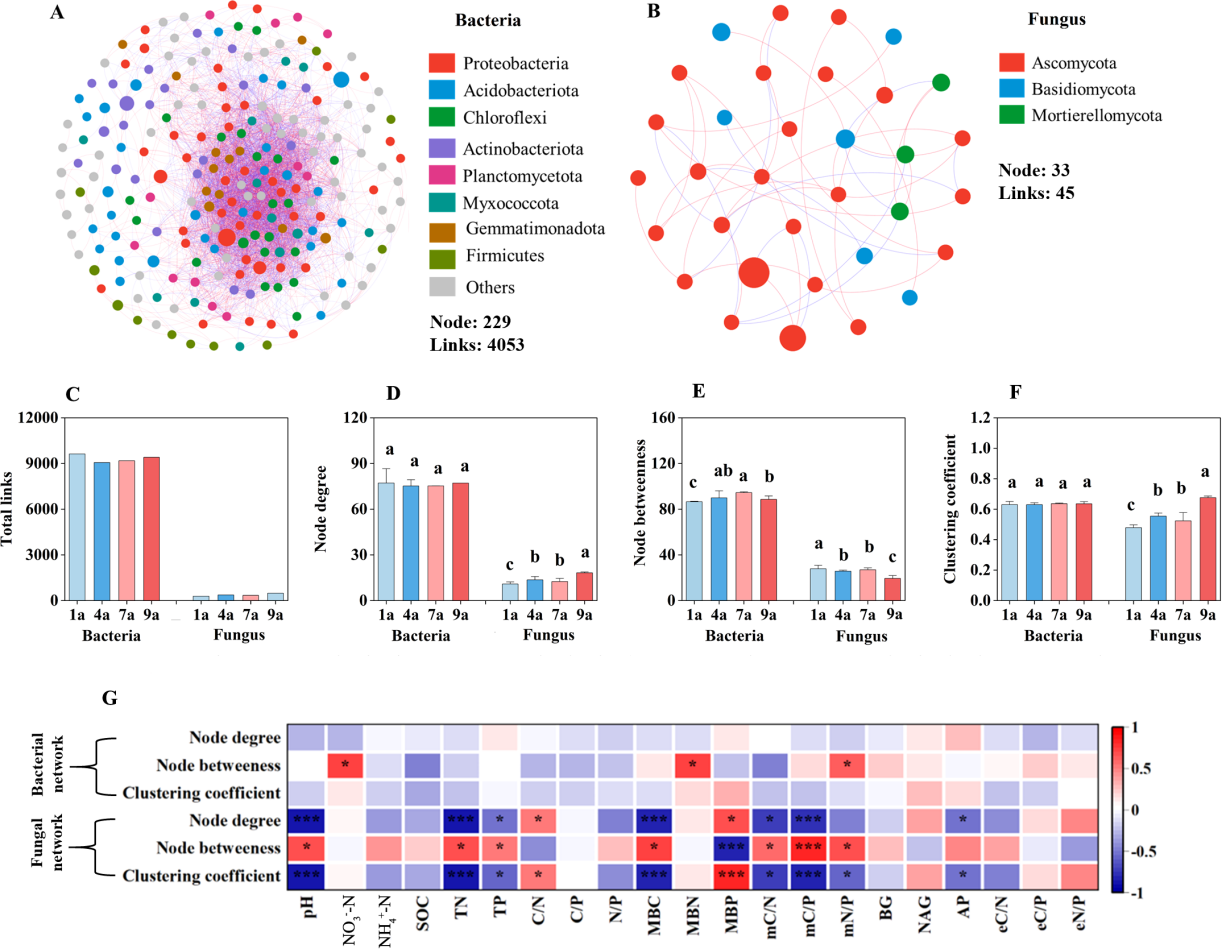
The co-occurrence networks of soil bacterial **(A)** and fungal **(B)** communities; parameters characterizing the complexity of networks **(C–E)**; correlations between soil chemical and biochemical properties and network complexity of soil bacteria and fungi **(F)**. NO_3_^–^-N, Nitrate nitrogen; NH_4_^+^-N, Ammonium nitrogen; SOC, soil organic carbon; TN, Total nitrogen; TP, Total phosphorus; C/N ratio, ratio of SOC and TN; C/P ratio, ratio of SOC and TP; N/P ratio, ratio of TN and TP; MBC, Microbial biomass carbon; MBN, Microbial biomass nitrogen; MBP, Microbial biomass phosphorus; mC/N ratio, ratio of MBC and MBN; mC/P ratio, ratio of MBC and MBP; mN/P ratio, ratio of MBN and MBP; BG, β-glucosidase; NAG, N-acetyl-β-D-glucosaminidase; AP, acid phosphomonoesterase; eC/N ratio, ratio of BG and NAG; eC/P ratio, ratio of BG and AP; eN/P ratio, ratioof NAG and AP. Different lowercase letters above the bar plots indicate significant differences at *P* < 0.05. Significant levels in correlation analyses: *P < 0.05; **P < 0.01; ***P < 0.001.

Distinct co-occurrence patterns were demonstrated for both bacterial and fungal soil communities in different stand ages of plantation (**Figure 4**). There were no significant differences in the node number of the bacterial and fungal communities between different stand ages of poplar plantation. In general, the link numbers and average degree of the bacterial communities decreased with the increase of stand age of poplar plantation, and the link numbers and average degree of the fungal communities increased with the increase of stand age of poplar plantation.

The node and link numbers were respectively greater in the bacterial communities (229 and 4053) than those in the fungal communities (33 and 45) (**Figures 4A, B**). The node degree and clustering coefficient of the bacterial communities did not show significant differences among 4 stand ages of poplar plantation (**Figures 4D, F**). The node betweenness of the bacterial communities in the 1-year-old plantation was higher than those in the other stand ages of poplar plantation (**Figure 4E**). No significant differences in the node betweenness of the bacterial communities were found between 4- and 7-year-old plantations. The node betweenness of the bacterial communities showed significant positive correlation with soil NO_3_^–^N concentration, MBN and mN/P ratio (**Figure 4G**). The node degree and clustering coefficient of the fungal communities in the 9-year-old plantation were higher than those in the other stand age of plantation (**Figures 4D, F**). There were no significant differences in the node degree, clustering coefficient and node betweenness of the fungal communities between 4- and 7-year-old plantations. The node degree, clustering coefficient and node betweenness of the fungal communities had significant relationship with soil N and P concentrations, MBC, MBP, mC/N ratio and mC/P ratio (**Figure 4G**).

### 3.4 Effects of soil chemical and biochemical properties on soil microbial diversity and community

The Shannon and Chao1 indexes of soil bacteria showed significant positive correlations with soil pH, total N concentration, MBC, mC/N ratio, mC/P ratio and eC/N ratio (**Table 2**). The Shannon and Chao1 indexes of soil bacteria showed significant negative correlations with soil MBP. The Chao1 index of soil fungi showed significant negative correlations with soil NO_3_^–^N concentration, MBN, mC/N ratio and BG. Furthermore, according to the RDA results, soil bacterial diversity showed significant relationship with soil microbial biomass, microbial stoichiometry, enzyme activity and enzyme stoichiometry (*P* ≤ 0.001; **Table 3**). These four variables explained 1.49%, 10.28%, 5.73% and 7.08% of variance of bacterial diversity, respectively. Therefore, the influence of microbial stoichiometry on soil bacterial diversity was more important than soil microbial biomass, enzyme activity and enzyme stoichiometry. Meanwhile, soil fungal diversity showed significant relationship with soil microbial biomass and enzyme activity (*P* < 0.05; **Table 3**). These two variables explained 6.62% and 9.81% of variance of fungal diversity, respectively.

**Table 2:** Correlations between soil microbial diversity and soil chemical and biochemical properties in the poplar plantations.

Chemical and biochemical properies	Bacteria			Fungi		
	Shannon	Chao1	Observed species	Shannon	Chao1	Observed species
pH	0.709**	0.780**	0.780^**^	0.167	0.078	0.328
NO_3_^–^N	-0.113	0.092	0.091	-0.274	-0.792**	-0.698^**^
NH_4_^+^-N	0.343	0.294	0.294	0.089	0.276	0.246
SOC	0.351	0.235	0.237	0.212	0.560*	0.544^*^
TN	0.643**	0.665**	0.665^**^	0.371	0.354	0.629^**^
TP	0.291	0.354	0.354	0.205	0.264	0.453
C/N	-0.256	-0.402	-0.401	-0.184	0.223	-0.038
C/P	0.138	0.032	0.033	0.042	0.170	0.084
N/P	0.435	0.384	0.384	0.186	0.082	0.174
MBC	0.687**	0.714**	0.714^**^	0.268	0.122	0.325
MBN	-0.251	-0.102	-0.103	-0.135	-0773**	-0.655^**^
MBP	-0.637**	-0.667**	-0.667^**^	-0.221	-0.332	-0.442
mC/N	0.677**	0.569*	0.570^*^	0.324	0.716**	0.772^**^
mC/P	0.705**	0.722**	0.722^**^	0.297	0.308	0.451
mN/P	0.393	0.495	0.495	0.143	-0.169	-0.018
BG	0.292	0.308	0.308	-0.031	-0.526*	-0.376
NAG	-0.356	-0.332	-0.332	-0.089	-0.396	-0.466
AP	0.303	0.257	0.257	0.358	0.171	0.372
eC/N	0.522*	0.520*	0.519^*^	0.065	-0.098	0.071
eC/P	0.047	0.082	0.082	-0.274	-0.457	-0.491
eN/P	-0.355	-0.317	-0.317	-0.206	-0.308	-0.444

NO_3_^–^-N, Nitrate nitrogen; NH4+-N, Ammonium nitrogen; SOC, soil organic carbon; TN, Total nitrogen; TP, Total phosphorus; C/N ratio, ratio of SOC and TN; C/P ratio, ratio of SOC and TP; N/P ratio, ratio of TN and TP; MBC, Microbial biomass carbon; MBN, Microbial biomass nitrogen; MBP, Microbial biomass phosphorus; mC/N ratio, ratio of MBC and MBN; mC/P ratio, ratio of MBC and MBP; mN/P ratio, ratio of MBN and MBP; BG, β-glucosidase; NAG, N-acetyl-β-D-glucosaminidase; AP, acid phosphomonoesterase; eC/N ratio, ratio of BG and NAG; eC/P ratio, ratio of BG and AP; eN/P ratio, ratio of NAG and AP; *P < 0.05; **P < 0.01; ***P < 0.001.

**Table 3:** RDA results of effects of soil chemical and biochemical properties on soil microbial diversity and community. .

Microbial diversity and community		Chemical properties		Chemical stoichiometry		Microbial biomass		Microbial stoichiometry		Enzyme activity		Enzyme stoichiometry	
		Exp (%)	*P*	Exp (%)	*P*	Exp (%)	*P*	Exp (%)	*P*	Exp (%)	*P*	Exp (%)	*P*
Bacteria	Diversity	0.63	0.431	0.25	0.615	1.49	**0.014**	**10.28**	**0.003**	5.73	**0.022**	7.08	**0.015**
	Composition	**52.8**	**0.001**	27.75	**0.001**	5.89	**0.007**	6.96	**0.005**	0.87	0.376	1.71	0.194
	Network	1.80	0.202	0.79	0.363	21.05	**0.001**	**25.16**	**0.001**	10.75	**0.007**	6.06	**0.013**
Fungi	Diversity	0.27	0.633	0.10	0.768	6.62	**0.015**	0.05	0.868	**9.81**	**0.003**	0.02	0.965
	Composition	2.32	0.118	**29.01**	**0.001**	2.06	0.132	7.90	**0.004**	0.70	0.505	0.28	0.768
	Network	1.80	0.170	0.79	0.385	21.05	**0.001**	**25.16**	**0.001**	10.75	**0.005**	6.07	**0.018**

Diversity, the diversity index (Shannon index, Chao1 index and observed species) of bacteria and fungi; Composition, the dominant phyla, classes and genera of bacteria and fungi; Network, the network parameters (Node degree, Node betweenness and Clustering coefficient) of bacteria and fungi; Exp, Variance explained; Bold indicated *P* < 0.05.

*Proteobacteria*, *Acidobacteriota*, *Firmicutes* and *Chloroflexi* showed significant correlations with eC/P and eN/P ratios (**Figure 2C**). *Actinobacteriota* had positive correlations with soil pH, soil NH_4_^+^-N concentration and N/P ratio. *Gammaproteobacteria* and *Alphaproteobacteria* negatively related to soil pH and N/P ratio. RB41 positively related to soil NO_3_^–^N concentration, P concentration and AP activity, and negatively related to soil NH_4_^+^-N concentration and C/N ratio. *Lactobacillus* showed positive relationship with soil pH, N concentration, eC/P ratio and eN/P ratio. *Ascomycota* showed a significant positive correlation with soil BG activity (**Figure 2D**). *Basidiomycota* negatively related to soil NO_3_^–^N concentration and MBN, and positively related to C/N ratio. *Mortierellomycota*, *Mortierellomycetes* and *Mortierella* negatively related to soil NH_4_^+^-N concentration and SOC. Besides, according to the RDA results, soil bacterial community composition showed significant relationship with soil chemical properties, chemical stoichiometry, microbial biomass and microbial stoichiometry (*P* ≤ 0.001; **Table 3**). These four variables explained 52.8%, 27.75%, 5.89% and 6.96% of variance of bacterial community composition, respectively. Thus, the influence of soil chemical properties and chemical stoichiometry on soil bacterial community composition were more important than microbial biomass and microbial stoichiometry. Meanwhile, soil fungal community composition showed significant relationship with soil chemical stoichiometry and microbial stoichiometry. These two variables explained 29.01% and 7.9% of variance of fungal community composition, respectively.

Soil bacterial network parameters showed significant relationship with soil microbial biomass, microbial stoichiometry, enzyme activity and enzyme stoichiometry (*P* ≤ 0.001; **Table 3**). These four variables explained 21.05%, 25.16%, 10.75% and 6.06% of variance of bacterial network parameters, respectively. Meanwhile, soil fungal network parameters showed significant relationship with soil microbial biomass, microbial stoichiometry, enzyme activity and enzyme stoichiometry. These four variables explained 21.05%, 25.16%, 10.75% and 6.07% of variance of fungal network parameters, respectively. Therefore, the influence of soil microbial biomass and microbial stoichiometry on soil bacterial and fungal network parameters were more important than enzyme activity and enzyme stoichiometry.

### 3.5 Response ratio of soil microbial community diversity and composition to stand age

**Figure 5:**
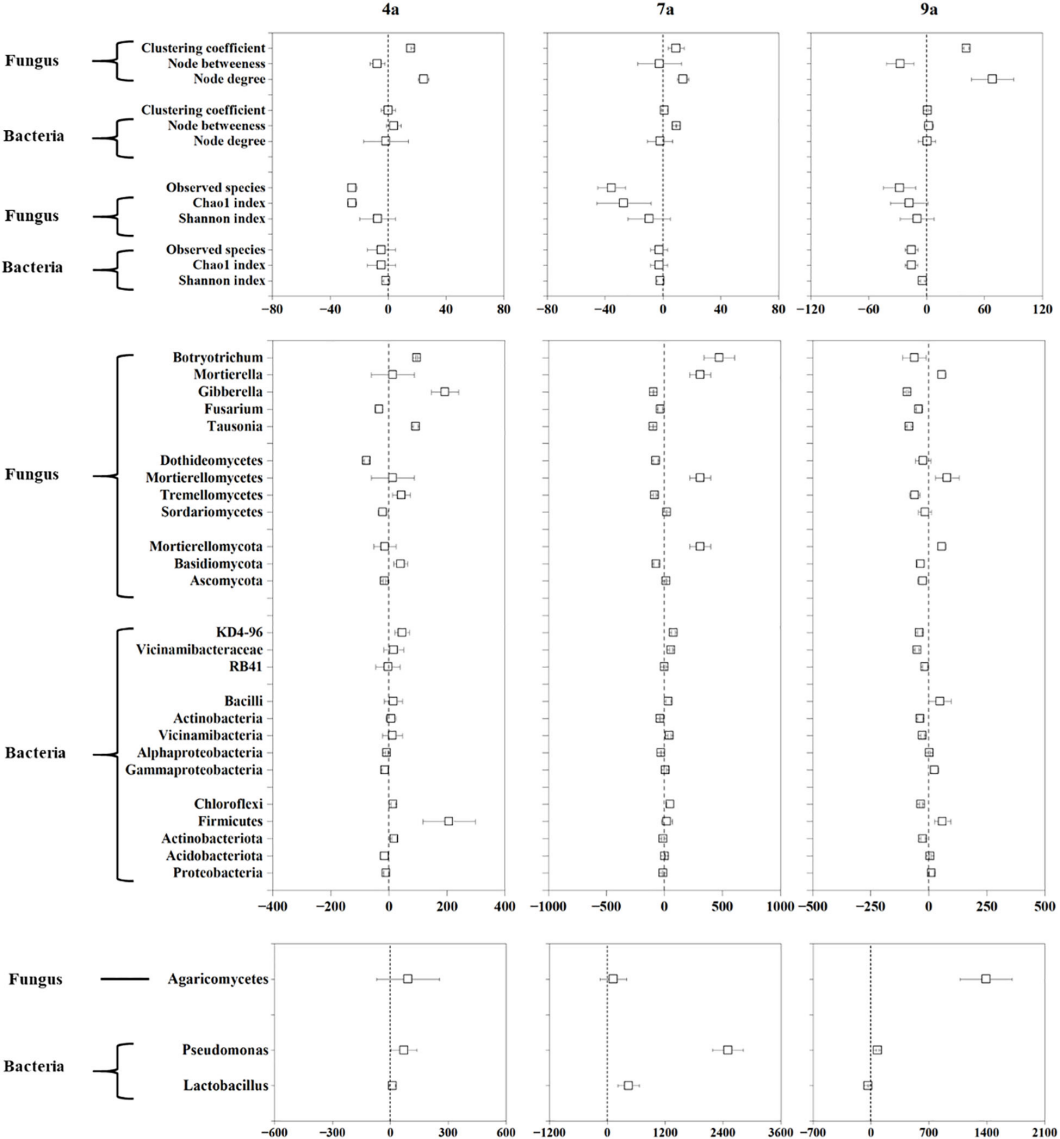
Response ratio (RR) of soil microbial diversity, network parameters and dominant phyla, classes and genera of bacteria and fungi at different stand ages.

The mean soil microbial RR varied markedly, ranging from -15.69% to -1.49% for soil bacterial diversity indexes, from -35.51% to -7.32% for soil fungal diversity indexes (**Figure 5**). The response ratio of node degree and node betweenness ranged from -1.35% to 9.23% for soil bacterial communities, and from -27.39% to 68.41% for soil fungal communities. Generally, the mean RR values of diversity indexes of bacterial and fungal communities were negative following primary stand age to the other three stand ages of poplar plantation. Additionally, compared to 1-year-old plantation, there was more prevalence of negative effects of 7-year-old plantation, and there was more prevalence of positive effects of 4- and 9-year-old plantations on soil fungal community.

## 4 Discussion

Globally, a few studies have highlighted the effects of stand age on soil chemical and biochemical properties and their stoichiometries. Previous studies found that SOC and MBC increased successively from young to mature stands and then declined for old stands (Wen et al., 2014; Xu et al., 2021). In a study of Chinese cypress forest stands, Gong et al. (2022) found lower active carbon and total nitrogen contents in overmature stands because of the lower amount of litter fall and poor litter quality. Similarly, the present study found that SOC, MBC and N concentration in the 9-year-old plantation were significantly lower than those in 1- and 4-year-old plantations. With stand development, canopy expansion reached maximum, the litterfall, and fine root production increase, resulting in more accumulation of organic matter (Sharma et al., 2009; Luan et al., 2010; He et al., 2021; Liu et al., 2022). However, in old stand age, fine root biomass and litterfall decreased because of less requirement of nutrients (He et al., 2021), which may result in lower SOC and MBC. Furthermore, the present findings confirmed that the older poplar plantation (9-year-old plantation) exhibited lower stoichiometry of chemical properties, microbial biomass and enzyme activity (i.e., C/P ratio, N/P ratio, mC/P ratio, mN/P ratio, eC/P ratio, eN/P ratio except for C/N ratio and eC/N ratio; **Figure 1**) compared to the young plantation (4-year-old plantation or 1-year-old plantation), which was consistent with our first hypothesis. It indicated that younger stands make favorable conditions for the growth of soil microbes; however, in old stage, the decrease in litter quantity and quality may reduce the growth of soil microbial community. Differently, Zhao et al. (2024) suggested that the older walnut orchards contained more C resources vs. N and P resources (as indicated by the C/N and C/P values) compared to the younger walnut orchards.

Our findings in the present study indicated that the diversity and composition of both bacteria and fungi did not increase with stand age, which is contrary to our second hypothesis. Compared with other stand ages, the Chao1 index and observed species of bacteria decreased in the 9-year-old plantation, and Chao1 index and observed species of fungi decreased in the 7-year-old plantation. Moreover, compared to 1-year-old plantation, there was more prevalence of negative effects of 4-, 7- and 9-year-old plantations on soil microbial diversity (**Figure 4**). Thus, soil microbial diversity decreased with increased stand ages of poplar plantation. This is mainly due to factors such as the microbial biomass and enzyme activity and their stoichiometry rather than chemical properties and chemical stoichiometry, which is contrary to the findings of Wang et al. (2021b) and Chen et al. (2016). For instance, the results of present study showed that the soil C/N ratio of microbial biomass and enzyme activity significantly related to soil Chao1 index and observed species of bacteria, and soil MBN and C/N ratio of microbial biomass significantly related to soil Chao1 index and observed species of fungi. Additionally, the stoichiometry of microbial biomass had greater influences on soil bacterial diversity compared to soil microbial biomass, enzyme activity and stoichiometry of enzyme activity according to the RDA results. Differently, soil enzyme activity showed greater effects on fungal diversity than the stoichiometry of microbial biomass.

In the present study, the most important bacterial species were *Proteobacteria*, *Acidobacteriota*, *Actinobacteriota*, *Firmicutes* and *Chloroflexi*. *Proteobacteria* always accounted for a larger proportion in bacterial community, similar to the findings of Bi et al. (2022). Five classes (*Gammaproteobacteria*, *Alphaproteobacteria*, *Vicinamibacteria*, *Actinobacteria* and *Bacilli*) of *Proteobacteria* were observed in the present study. Many important taxa belong to *Gammaproteobacteria*, such as *Pseudomonadaceae*, which is widely distributed in soil and plants and have a strong ability to decompose organic matter and can use a variety of organic matter as energy sources. The 9-year-old plantation had the greater relative abundance of *Proteobacteria*, *Gammaproteobacteria* and *Pseudomonadaceae*, which indicated that the soil microbes were more active and had higher substrate decomposition efficiency during this growth period. The fast-growing, copiotrophic members of the phylum *Proteobacteria* are capable of utilizing various C substrates (Fierer et al., 2007) and positively correlated with the soil organic C, N fractions, and available nutrients (Qiu et al., 2021). Similarly, the relative abundance of *Proteobacteria* showed significant relationship with C/P ratio of microbial biomass and enzyme activity and N/P ratio of enzyme activity in the present study. Meanwhile, the relative abundance of *Gammaproteobacteria* showed significant relationship with soil NO_3_^–^-N concentration, NH_4_^+^-N concentration and P concentration. Besides, chemical properties rather than chemical stoichiometry, soil microbial biomass and enzyme activity showed significant effects on soil microbial community composition. Therefore, the markedly increased *Proteobacteria* abundance in the 9-year-old plantation compared to the other stand ages of plantation may partially be ascribed to the changed soil chemical properties.

The fungal community was mainly composed of *Ascomycota*, *Basidiomycota* and *Mortierellomycota*, which was consistent with the previous studies (Kernaghan and Patriquin, 2011; Toju et al., 2013). Bi et al. (2022) found that the proportion of fungal community structure in the rhizosphere showed strong heterogeneity among plantations with various forest ages, and the reasons for fungal community changes may be caused by the plant growth conditions, root changes, and root exudates. Moreover, compared to 1-year-old plantation, there was more prevalence of negative effects of 7-year-old plantation, and there was more prevalence of positive effects of 4- and 9-year-old plantation on soil fungal community (**Figure 4**). Thus, the composition of bacteria and fungi varied with the different stand ages in the present study. The impact of stand age on soil microbial community composition is specific and stage-dependent, rather than following a simple linear trend with increasing stand age. Compared with young plantations, mature plantations have obviously different requirements for nutrients which significantly related to the structure of the microbial community (Bi et al., 2022). Thus, changes of microbial community might be partly attributed to the differences in nutrient requirements of different aged forests (Goldfarb et al., 2011).

Co-occurrence network analysis is used to explain the microbial community aggregation, which can clearly show the network features and interrelationships among OTUs (Agler et al., 2016; Ma et al., 2016). The positive and negative correlation coefficients between two OTUs in the co-occurrence network represent co-aggregation and mutual exclusion, respectively (Faust and Raes, 2012). In the present study, there were no significant differences in node degree and clustering coefficient of bacteria among different stand ages of poplar plantation, while the node degree and clustering coefficient of fungal communities in the 9-year-old plantation were higher than those in the other stand ages. Thus, the complexity of the co-occurrence network of bacteria was not significantly changed with stand age, while the complexity of the co-occurrence network of fungi gradually increased with stand age, which is contrary to our second hypothesis. It indicated that the effect of aggregation or repulsion of the fungi taxon increased as the plantation became older, contrary to a previous study of Bi et al. (2022). Additionally, the results of RDA showed that soil microbial biomass and its stoichiometry had significant effects on the co-occurrence network of fungi compared to soil chemical and biochemical properties and enzyme activity, which is contrary to our third hypothesis. The node degree and clustering coefficient were significantly negatively related to soil MBC and C/N and C/P ratios of microbial biomass. Thus, the increasing network complexity of fungal community in the 9-year-old plantation might be attributed to the decreased soil microbial biomass and its stoichiometry compared to that in the other stand ages of plantation. Furthermore, the present results suggested that soil bacterial and fungal networks had diverse characteristics and responses to different stand ages of poplar plantation, with stand age having smaller effects on bacterial networks than on fungal networks, contrary to previous studies (Liu et al., 2024). Therefore, compared with fungal networks, bacterial networks are more stable and less prone to change with stand age of poplar plantation.

## 5 Conclusions

In general, our results suggest that the 9-year-old plantation exhibited lower chemical properties, microbial biomass and stoichiometry of chemical properties, microbial biomass and enzyme activity compared to the younger plantation (4-year-old plantation or 1-year-old plantation). Changes in the diversity and compositions of both soil bacterial and fungal communities were associated with stand ages of plantation. Compared to 1-year-old plantation, there was more prevalence of negative effects of 4-, 7- and 9-year-old plantations on soil microbial diversity. Thus, soil microbial diversity in 4-, 7- and 9-year-old plantations was lower than those in the 1-year-old plantation. This is mainly due to factors such as the microbial biomass and enzyme activity and their stoichiometry rather than chemical properties and chemical stoichiometry. The most important bacterial species were *Proteobacteria*, *Acidobacteriota*, *Actinobacteriota*, *Firmicutes* and *Chloroflexi*, and the fungal community was mainly composed of *Ascomycota*, *Basidiomycota* and *Mortierellomycota*. The changes in bacterial community compositions might be partly attributed to soil chemical properties and chemical stoichiometries rather than to soil microbial biomass, enzyme activity and their stoichiometries, and the changes in fungal community compositions might be partly attributed to soil chemical stoichiometry rather than soil chemical properties, microbial biomass, enzyme activity and their stoichiometry. Moreover, the complexity of the co-occurrence network of bacteria was not significantly changed with stand age, while the complexity of the co-occurrence network of fungi gradually increased with stand age. Thus, compared with fungal networks, bacterial networks are more stable and less prone to change with stand age of poplar plantation. Soil microbial biomass and its stoichiometry showed the greater influences on the co-occurrence network of bacteria and fungi compared to soil chemical properties, enzyme activity and their stoichiometry. Taken together, these results suggest that the results of present study found that the impact of stand age on soil microbial community diversity and composition is specific and stage-dependent, rather than following a simple linear trend with increasing age, and this may be due to the influence of stand age on stoichiometry of soil microbial biomass.

## Data Availability

The data that support the findings of this study are available from the corresponding author upon reasonable request.

## References

[B1] Agler M. T. Ruhe J. Kroll S. Morhenn C. Kim S. T. Weigel D. . (2016). Microbial hub taxa link host and abiotic factors to plant microbiome variation. PloS Biol. 14, e1002352. doi: 10.1371/journal.pbio.1002352, PMID: 26788878 PMC4720289

[B2] Bach C. E. Warnock D. D. Van Horn D. J. Weintraub M. N. Sinsabaugh R. L. Allison S. D. . (2013). Measuring phenol oxidase and peroxidase activities with pyrogallol, L-DOPA, and ABTS: Effect of assay conditions and soil type. Soil Biol. Biochem. 67, 183–191. doi: 10.1016/j.soilbio.2013.08.022

[B3] Bi B. Yuan Y. Zhang H. Wu Z. Wang Y. Han F. (2022). Rhizosphere soil metabolites mediated microbial community changes of *Pinus sylvestris* var. *mongolica* across stand ages in the Mu Us Desert. Appl. Soil Ecol. 169, 104222. doi: 10.1016/j.apsoil.2021.104222

[B4] Cao Y. S. Fu S. L. Zou X. M. Cao H. L. Shao Y. H. Zhou L. X. (2010). Soil microbial community composition under *Eucalyptus* plantations of different age in subtropical China. Eur. J. Soil Biol. 46, 128–135. doi: 10.1016/j.ejsobi.2009.12.006

[B5] Chen X. L. Chen H. Y. H. Chen X. Wang J. Chen B. Wang D. . (2016). Soil labile organic carbon and carbon-cycle enzyme activities under different thinning intensities in Chinese fir plantations. Appl. Soil Ecol. 107, 162–169. doi: 10.1016/j.apsoil.2016.05.016

[B6] Chen W. Wang J. Chen X. Meng Z. Xu R. Duoji D. . (2022). Soil microbial network complexity predicts ecosystem function along elevation gradients on the Tibetan Plateau. Soil Biol. Biochem. 172, 108766. doi: 10.1016/j.soilbio.2022.108766

[B7] Chen F. Zheng H. Zhang K. Ouyang Z. Lan J. Li H. . (2013). Changes in soil microbial community structure and metabolic activity following conversion from native *Pinus massoniana* plantations to exotic *Eucalyptus* plantations. For. Ecol. Manage. 291, 65–72. doi: 10.1016/j.foreco.2012.11.016

[B8] Deng Y. Jiang Y. H. Yang Y. He Z. Luo F. Zhou J. (2012). Molecular ecological network analyses. BMC Bioinf. 13, 1–20. doi: 10.1186/1471-2105-13-113, PMID: 22646978 PMC3428680

[B9] Dixon P. (2003). VEGAN, a package of R functions for community ecology. J. Veg. Sci. 14, 927–930. doi: 10.1111/j.1654-1103.2003.tb02228.x

[B10] Faust K. Raes J. (2012). Microbial interactions: from networks to models. Nat. Rev. Microbiol. 10, 538–550. doi: 10.1038/nrmicro2832, PMID: 22796884

[B11] Fierer N. Bradford M. A. Jackson R. B. (2007). Toward an ecological classification of soil bacteria. Ecology 88, 1354–1364. doi: 10.1890/05-1839, PMID: 17601128

[B12] Frey S. D. Knorr M. Parrent J. L. Simpson R. T. (2004). Chronic nitrogen enrichment affects the structure and function of the soil microbial community in temperate hardwood and pine forests. For. Ecol. Manage. 196, 159–171. doi: 10.1016/j.foreco.2004.03.018

[B13] German D. P. Marcelo K. R. B. Stone M. M. Allison S. D. (2012). The Michaelis-Menten kinetics of soil extracellular enzymes in response to temperature: A cross-latitudinal study. Global Change Biol. 18, 1468–1479. doi: 10.1111/j.1365-2486.2011.02615.x

[B14] Goldfarb K. C. Karaoz U. Hanson C. A. Santee C. A. Bradford M. A. Treseder K. K. . (2011). Differential growth responses of soil bacterial taxa to carbon substrates of varying chemical recalcitrance. Front. Microbiol. 2, 94. doi: 10.3389/fmicb.2011.00094, PMID: 21833332 PMC3153052

[B15] Gong X. Jarvie S. Wen J. Su N. Yan Y. Liu Q. . (2024). Compared with soil fungal diversity and microbial network complexity, soil bacterial diversity drives soil multifunctionality during the restoration process. J. Environ. Manage. 354, 120379. doi: 10.1016/j.jenvman.2024.120379, PMID: 38368806

[B16] Gong S. Yang X. Xu X. Lai Y. Zhang Z. Kong Y. (2022). Effect of stand age on the temporal dynamics of soil active carbon and nitrogen in Chinese cypress artificial forests. Soil Sci. Plant Nutr. 68, 64–71. doi: 10.1080/00380768.2022.2030194

[B17] Guo Q. F. Ren H. (2014). Productivity as related to diversity and age in planted versus natural forests. Glob. Ecol. Biogeogr. 23, 1461–1471. doi: 10.1111/geb.12238

[B18] Han Y. Jin S. Chen W. Zhan M. Yuan Z. Wang X. . (2022). Biophysical controls on energy exchange and water use efficiency over a poplar plantation in Northern Hilly China. Agr. Water Manage. 273, 107920. doi: 10.1016/j.agwat.2022.107920

[B19] He X. Huang Y. Zhang Q. Ye S. Wang S. (2021). Distribution of organic carbon fractions in soil aggregates in Chinese fir plantations with different stand ages. Ecol. Process. 10, 49. doi: 10.1186/s13717-021-00321-5, PMID: 35543045

[B20] Hill B. H. Elonen C. M. Seifert L. R. May A. A. Tarquinio E. (2012). Microbial enzyme stoichiometry and nutrient limitation in us streams and rivers. Ecol. Indic. 18, 540–551. doi: 10.1016/j.ecolind.2012.01.007

[B21] Inagaki Y. Miura S. Kohzu A. (2004). Effects of forest type and stand age on litterfall quality and soil N dynamics in Shikoku district, southern Japan. For. Ecol. Manage. 202, 107–117. doi: 10.1016/j.foreco.2004.07.029

[B22] Kempers A. J. Zweers A. (1986). Ammonium determination in soil extracts by the salicylate method. Commun. Soil Sci. Plan. 17, 715–723. doi: 10.1080/00103628609367745

[B23] Kernaghan G. Patriquin G. (2011). Host associations between fungal root endophytes and boreal trees. Microb. Ecol. 62, 460–473. doi: 10.1007/s00248-011-9851-6, PMID: 21475991

[B24] Li S. Huang X. Tang R. Li J. Zhu B. Su J. (2023). Soil microbial diversity and network complexity sustain ecosystem multifunctionality following afforestation in a dry-hot valley savanna. Catena 231, 107329. doi: 10.1016/j.catena.2023.107329

[B25] Li J. Zhou X. Yan J. Li H. He J. (2015). Effects of regenerating vegetation on soil enzyme activity and microbial structure in reclaimed soils on a surface coal mine site. Appl. Soil Ecol. 87, 56–62. doi: 10.1016/j.apsoil.2014.11.010

[B26] Liu R. He Y. Du Z. Zhou G. Zhou L. Wang X. . (2022). Root production and microbe-derived carbon inputs jointly drive rapid soil carbon accumulation at the early stages of forest succession. Forests 13, 2130. doi: 10.3390/f13122130

[B27] Liu M. Lv X. Zhang W. Jiang M. Tian L. Qin L. . (2024). Biological interactions control bacterial but not fungal β diversity during vegetation degradation in saline–alkaline soil. Sci. Total Environ. 919, 170826. doi: 10.1016/j.scitotenv.2024.170826, PMID: 38340840

[B28] Luan J. Xiang C. Liu S. Luo Z. Gong Y. Zhu X. (2010). Assessments of the impacts of Chinese fir plantation and natural regenerated forest on soil organic matter quality at Longmen mountain, Sichuan, China. Geoderma 156, 228–236. doi: 10.1016/j.geoderma.2010.02.021

[B29] Ma B. Wang H. Dsouza M. Lou J. He Y. Dai Z. M. . (2016). Geographic patterns of co-occurrence network topological features for soil microbiota at continental scale in eastern China. ISME J. 10, 1891–1901. doi: 10.1038/ismej.2015.261, PMID: 26771927 PMC5029158

[B30] Moreira A. Fageria N. K. Garcia A. Garcia Y. (2011). Soil fertility, mineral nitrogen, and microbial biomass in upland soils of the central Amazon under different plant covers. Commun. Soil Sci. Plant Anal. 42, 694–705. doi: 10.1080/00103624.2011.550376

[B31] Morriën E. Hannula S. E. Snoek L. B. Helmsing N. R. Zweers H. De Hollander M. (2017). Soil networks become more connected and take up more carbon as nature restoration progresses. Nat. Commun. 8, 14349. doi: 10.1038/ncomms14349, PMID: 28176768 PMC5309817

[B32] Nakayama M. Imamura S. Taniguchi T. Tateno R. (2019). Does conversion from natural forest to plantation affect fungal and bacterial biodiversity, community structure, and co-occurrence networks in the organic horizon and mineral soil? For. Ecol. Manage. 446, 238–250. doi: 10.1016/j.foreco.2019.05.042

[B33] Nannipieri P. Pedrazzini F. Arcara P. G. Piovanelli C. (1979). Changes in amino acids, enzyme activities, and biomasses during soil microbial growth. Soil Sci. 127, 26–34. doi: 10.1097/00010694-197901000-00004

[B34] National Forestry and Grassland Administration (2020). Seventh national forest resource inventory report, (2014-2018). For. Resour. Manage. 1-2.

[B35] Nelson D. W. Sommers L. E. (1996). “ Total carbon, organic carbon and organic matter,” in Methods of Soil Analysis, Part 3: Chemical Methods. Eds. Sparks D. L. Page A. L. Helmke P. A. Loeppert R. H. Soltanpour P. N. Tabatabai M. A. Johnston C. T. Sumner M. E. ( Soil Science Society of America, Wisconsin), 961–1010.

[B36] Ni H. Su W. Fan S. Chu H. (2021). Effects of intensive management practices on rhizosphere soil properties, root growth, and nutrient uptake in Moso bamboo plantations in subtropical China. For. Ecol. Manage. 493, 119083. doi: 10.1016/j.foreco.2021.119083

[B37] Pajares S. Bohannan B. J. (2016). Ecology of nitrogen fixing, nitrifying, and denitrifying microorganisms in tropical forest soils. Front. Microbiol. 7, 1045. doi: 10.3389/fmicb.2016.01045, PMID: 27468277 PMC4932190

[B38] Polyanskaya L. M. Stepanov A. L. Chakmazyan K. V. (2017). The impact of hydrogen emission on the structure of soil microbial biomass. Eurasian Soil Sci. 50, 57–63. doi: 10.1134/S1064229317010112

[B39] Qiang W. Yang B. Liu Y. Qi K. B. Yang T. H. Pang X. Y . (2020). Effects of reclamation age on soil microbial communities and enzymatic activities in the sloping citrus orchards of southwestern China. Appl. Soil Ecol. 152, 103566. doi: 10.1016/j.apsoil.2020.103566

[B40] Qiu L. Zhang Q. Zhu H. Reich P. B. Banerjee S. van der Heijden M. G. A. . (2021). Erosion reduces soil microbial diversity, network complexity and multifunctionality. ISME J. 15, 2474–2489. doi: 10.1038/s41396-021-00913-1, PMID: 33712698 PMC8319411

[B41] Ross D. J. Sparling G. P. Burke C. M. Smith C. T. (1995). Microbial biomass C and N, and mineralizable-N, in litter and mineral soil under *Pinus radiata* on a coastal sand: influence of stand age and harvest management. Plant Soil 175, 167–177. doi: 10.1007/BF00011352

[B42] Sengupta A. Dick W. A. (2017). Methanotrophic bacterial diversity in two diverse soils under varying land-use practices as determined by high-throughput sequencing of the *pmoA* gene. Appl. Soil Ecol. 119, 35–45. doi: 10.1016/j.apsoil.2017.05.031

[B43] Sharma G. Sharma R. Sharma E. (2009). Impact of stand age on soil C, N and P dynamics in a 40-year chronosequence of alder-cardamom agroforestry stands of the Sikkim Himalaya. Pedobiologia 52, 401–414. doi: 10.1016/j.pedobi.2009.01.003

[B44] Sheng H. Zhou P. Zhang Y. Kuzyakov Y. Zhou Q. Ge T. . (2015). Loss of labile organic carbon from subsoil due to land-use changes in subtropical China. Soil Biol. Biochem. 88, 148–157. doi: 10.1016/j.soilbio.2015.05.015

[B45] Shi X. Yang J. Cui H. Song W. Zhang P. Bi X. (2024). Effects of long-term drainage and afforestation on carbon utilization function of soil microbial communities vary between the types of subtropical moss peatlands. Glob. Ecol. Conserv. 49, e02803. doi: 10.1016/j.gecco.2024.e02803

[B46] Song Y. Y. Song C. C. Yang G. S. Miao Y. Q. Wang J. Y. Guo Y. D. (2012). Changes in labile organic carbon fractions and soil enzymes activities after marshland reclamation and restoration in the Sanjiang plain in Northeast China. Environ. Manage. 50, 418–426. doi: 10.1007/s00267-012-9890-x, PMID: 22744158

[B47] Song L. Zhu J. Zhang T. Wang K. Wang G. Liu J. (2021). Higher canopy transpiration rates induced dieback in poplar (*Populus* × *xiaozhuanica*) plantations in a semiarid sandy region of Northeast China. Agr. Water Manage. 243, 106414. doi: 10.1016/j.agwat.2020.106414

[B48] Tisdall J. M. (1994). Possible role of soil microorganisms in aggregation in soils. Plant Soil 159, 115–121. doi: 10.1007/BF00000100

[B49] Toju H. Yamamoto S. Sato H. Tanabe A. S. Gilbert G. S. Kadowaki K. (2013). Community composition of root-associated fungi in a *Quercus*-dominated temperate forest: "codominance" of mycorrhizal and root-endophytic fungi. Ecol. Evol. 3, 1281–1293. doi: 10.1002/ece3.546, PMID: 23762515 PMC3678483

[B50] Trogisch S. He J. S. Hector A. Scherer-Lorenzen M. (2016). Impact of species diversity, stand age and environmental factors on leaf litter decomposition in subtropical forests in China. Plant Soil 400, 337–350. doi: 10.1007/s11104-015-2737-5

[B51] Tu Q. Yan Q. Deng Y. Michaletz S. T. Buzzard V. Weiser M. D. . (2020). Biogeographic patterns of microbial cooccurrence ecological networks in six American forests. Soil Biol. Biochem. 148, 107897. doi: 10.1016/j.soilbio.2020.107897

[B52] Wang C. Q. Xue L. Dong Y. H. Jiao R. Z. (2021b). Effects of stand density on soil microbial community composition and enzyme activities in subtropical *Cunninghamia lanceolate* (Lamb.) Hook plantations. For. Ecol. Manage. 479, 118559. doi: 10.1016/j.foreco.2020.118559

[B53] Wang C. Xue L. Jiao R. (2021a). Soil organic carbon fractions, C-cycling associated hydrolytic enzymes, and microbial carbon metabolism vary with stand age in *Cunninghamia lanceolate* (Lamb.) Hook plantations. For. Ecol. Manage. 482, 118887. doi: 10.1016/j.foreco.2020.118887

[B54] Wen L. Lei P. Xiang W. Yan W. Liu S. (2014). Soil microbial biomass carbon and nitrogen in pure and mixed stands of *Pinus massoniana* and *Cinnamomum camphora* differing in stand age. For. Ecol. Manage. 328, 150–158. doi: 10.1016/j.foreco.2014.05.037

[B55] Wu B. Ding M. Zhang H. Devlin A. T. Wang P. Chen L. . (2023). Reduced soil multifunctionality and microbial network complexity in degraded and revegetated alpine meadows. J. Environ. Manage. 343, 118182. doi: 10.1016/j.jenvman.2023.118182, PMID: 37224687

[B56] Wu H. L. Xiang W. H. Ouyang S. Xiao W. F. Li S. G. Chen L. . (2020). Tree growth rate and soil nutrient status determine the shift in nutrient-use strategy of Chinese fir plantations along a chronosequence. For. Ecol. Manage. 460, 117896. doi: 10.1016/j.foreco.2020.117896

[B57] Xia Q. Chen L. Xiang W. H. Ouyang S. Wu H. L. Lei P. F. . (2021). Increase of soil nitrogen availability and recycling with stand age of Chinese-fir plantations. For. Ecol. Manage. 480, 118643. doi: 10.1016/j.foreco.2020.118643

[B58] Xu M. Jian J. Wang J. Zhang Z. Yang G. Han X. . (2021). Response of root nutrient resorption strategies to rhizosphere soil microbial nutrient utilization along *Robinia pseudoacacia* plantation chronosequence. For. Ecol. Manage. 489, 119053. doi: 10.1016/j.foreco.2021.119053

[B59] Xu Y. Li C. Zhu Y. Wang Z. Zhu W. Wu L. . (2022). The shifts in soil microbial community and association network induced by successive planting of Eucalyptus plantations. For. Ecol. Manage. 505, 119877. doi: 10.1016/j.foreco.2021.119877

[B60] Zhao Y. Yang W. Liu Y. Zhang X. Li Y. Qi G. . (2024). Linking soil organic carbon characteristics, nutrient stoichiometry, and microbial community to eco-enzymatic stoichiometry within aggregates in different aged walnut plantations. Eur. J. Soil Biol. 121, 103627. doi: 10.1016/j.ejsobi.2024.103627

[B61] Zhou J. Deng Y. Luo F. He Z. Tu Q. Zhi X. (2010). Functional molecular ecological networks. mBio 1, e00169–e00110. doi: 10.1128/mBio.00169-10, PMID: 20941329 PMC2953006

[B62] Zhou J. Zhang Z. Sun G. Fang X. Zha T. Mcnulty S. . (2013). Response of ecosystem carbon fluxes to drought events in a poplar plantation in Northern China. For. Ecol. Manage. 300, 33–42. doi: 10.1016/j.foreco.2013.01.007

[B63] Zhu L. Tang Y. Weng Y. Huang K. Wang J. Zhao J. . (2021). Effects of burning harvested residues on the archaeal and bacterial communities of *Eucalyptus urophylla* substituting native vegetation. Appl. Soil Ecol. 158, 103796. doi: 10.1016/j.apsoil.2020.103796

